# Supplementary material for: ‘Stolen Time’—Delivering Nursing at the Bottom of a Hierarchy: An Ethnographic Study of Barriers and Facilitators for Evidence-Based Nursing for Patients with Community-Acquired Pneumonia
Source: Healthcare (Basel). 2021 Nov 9;9(11):1524. doi: 10.3390/healthcare9111524 (PMC8620708; doi:10.3390/healthcare9111524)
Supplement: Supplementary file 1 [file healthcare-09-01524-s001.zip › healthcare-1440275-supplementary.pdf]

Additional File S1
Focus-group interview guide based on the TDF framework of 14 domains [26].

TDF DOMAIN	INTERVIEW QUESTIONS
**Knowledge**	1. Do you know which nursing care interventions are important for patients with CAP?<br><br>2. How well do you know EBG for CAP? Do you know, if they describe nursing interventions for patients with CAP?<br><br>*Researcher (SE) presents research results of performed nursing interventions in a unit [6].*<br><br>3. Do you have an idea, why these results look the way they do?
**Skills**	4. Have you been trained or received lectures in how to perform nursing interventions: *respiratory therapy, sputum mobilisation, oral care, mobilisation, fluid, and nutrition therapy?*<br><br>If YES, have training or lectures supported you to perform…
**Social/professional role and identity**	5. Who do you think is responsible for, that patients receive: *respiratory therapy, sputum mobilisation, oral care, mobilisation, fluid, and nutrition therapy?*
**Beliefs about capabilities + Memory, attention and decision processes**	6. Do you experience any difficulties to perform: *respiratory therapy, sputum mobilisation, oral care, mobilisation, fluid, and nutrition therapy?*<br><br>What makes it easy / difficult to do?<br><br>7. Are there negative aspects of performing: *respiratory therapy, sputum mobilisation, oral care, mobilisation, fluid, and nutrition therapy?*<br><br>8. Are there situations where you wittingly have decided not to perform: *respiratory therapy, sputum mobilisation, oral care, mobilisation, fluid, and nutrition therapy?*
**Beliefs about consequences**	9. Do you know why it is important and what are the consequences if patients do not receive: *respiratory therapy, sputum mobilisation, oral care, mobilisation, fluid, and nutrition therapy?*<br>10. Are there benefits or consequences for you or the unit, if the patient receive / do not receive: *respiratory therapy, sputum*

	mobilisation, oral care, mobilisation, fluid, and nutrition therapy?<br><br>If YES, what are the benefits and consequences?
**Environmental context and resources**	11. What are the barriers for performing: *respiratory therapy, sputum mobilisation, oral care, mobilisation, fluid, and nutrition therapy?*<br><br>*Ask about: Workflows, priorities, organisational resources, culture, physical conditions*
**Social influences**	12. To what extend are you influenced by your colleagues' performance regarding: *respiratory therapy, sputum mobilisation, oral care, mobilisation, fluid, and nutrition therapy?*<br><br>If YES, describe how.
**Emotion**	13. Does the psychological working environment (e.g. stress) affect your performance regarding: *respiratory therapy, sputum mobilisation, oral care, mobilisation, fluid, and nutrition therapy?*<br><br>If YES, describe how.
**Intentions**<br>+<br>**Optimism**	14. How often do you feel you have to give low priority to: *respiratory therapy, sputum mobilisation, oral care, mobilisation, fluid, and nutrition therapy?*
**Goals**	15. When, or in which situations do you succeed in performing these nursing interventions?<br><br>16. Are there any procedures or workflows that motivate you to perform these interventions?
**Behavioural regulation +**<br>**Reinforcement**	17. What will support you to perform nursing care interventions according to EBG?

Reference

[26]: Cane J, et al. Validation of the theoretical domains framework for use in behavior change and implementation research. Implementation Sci. 2012,7:37; doi:10.1186/1748-5908-7-37.

[6]: Eekholm S, Ahlstrom G, Kristensson J, Lindhardt T. Gaps between current clinical practice and evidence-based guidelines for treatment and care of older patients with Community Acquired Pneumonia: a descriptive cross-sectional study. BMC Infect Dis. 2020;20(1):73; doi:10.1186/s12879-019-4742-4.

Additional File S2
Standards for Reporting Qualitative Research (SRQR)*

http://www.equator-network.org/reporting-guidelines/srqr/

Title and abstract

	**Page/line no(s).**
**Title** - Concise description of the nature and topic of the study Identifying the study as qualitative or indicating the approach (e.g., ethnography, grounded theory) or data collection methods (e.g., interview, focus group) is recommended	1/1-5 (Title page)
**Abstract** - Summary of key elements of the study using the abstract format of the intended publication; typically includes background, purpose, methods, results, and conclusions	1/13-27

Introduction

**Problem formulation** - Description and significance of the problem/phenomenon studied; review of relevant theory and empirical work; problem statement	1-2/31-70
**Purpose or research questio**n - Purpose of the study and specific objectives or questions	1/39-41<br>2/59-62, 68-70

Methods

**Qualitative approach and research paradigm** - Qualitative approach (e.g., ethnography, grounded theory, case study, phenomenology, narrative research) and guiding theory if appropriate; identifying the research paradigm (e.g., postpositivist, constructivist/ interpretivist) is also recommended; rationale**	2-3/73-117
**Researcher characteristics and reflexivity** - Researchers' characteristics that may influence the research, including personal attributes, qualifications/experience, relationship with participants, assumptions, and/or presuppositions; potential or actual interaction between researchers' characteristics and the research questions, approach, methods, results, and/or transferability	2-3/74-101<br>6/208-237
**Context** - Setting/site and salient contextual factors; rationale**	4/119-156
**Sampling strategy** - How and why research participants, documents, or events were selected; criteria for deciding when no further sampling was necessary (e.g., sampling saturation); rationale**	4/142-156
**Ethical issues pertaining to human subjects** - Documentation of approval by an appropriate ethics review board and participant consent, or explanation for lack thereof; other confidentiality and data security issues	15-16/678-690
**Data collection methods** - Types of data collected; details of data collection procedures including (as appropriate) start and stop dates of data collection and analysis, iterative process, triangulation of sources/methods, and modification of procedures in response to evolving study findings; rationale**	4-6/157-207

**Data collection instruments and technologies** - Description of instruments (e.g., interview guides, questionnaires) and devices (e.g., audio recorders) used for data collection; if/how the instrument(s) changed over the course of the study	3-4/101-118<br>4-6/157-207
**Units of study** - Number and relevant characteristics of participants, documents, or events included in the study; level of participation (could be reported in results)	4/142-156
**Data processing** - Methods for processing data prior to and during analysis, including transcription, data entry, data management and security, verification of data integrity, data coding, and anonymization/de-identification of excerpts	6/208-237<br>15/683-688<br>16/691-693
**Data analysis** - Process by which inferences, themes, etc., were identified and developed, including the researchers involved in data analysis; usually references a specific paradigm or approach; rationale**	6/208-237
**Techniques to enhance trustworthiness** - Techniques to enhance trustworthiness and credibility of data analysis (e.g., member checking, audit trail, triangulation); rationale**	6/208-237<br>15/670-674, 15-16687-690

Results/findings

**Synthesis and interpretation** - Main findings (e.g., interpretations, inferences, and themes); might include development of a theory or model, or integration with prior research or theory	6-12/238-500
**Links to empirical data** - Evidence (e.g., quotes, field notes, text excerpts, photographs) to substantiate analytic findings	7/247-250<br>8/280-282<br>8/294-296<br>8/325-329<br>9/353-356<br>10/382-385<br>10/407-409<br>10/422-427<br>11/434-436<br>11/445-448<br>11/472-476<br>12/485-489

Discussion

**Integration with prior work, implications, transferability, and contribution(s) to the field -** Short summary of main findings; explanation of how findings and conclusions connect to, support, elaborate on, or challenge conclusions of earlier scholarship; discussion of scope of application/generalizability; identification of unique contribution(s) to scholarship in a discipline or field	12-15/501-660
**Limitations** - Trustworthiness and limitations of findings	14-15/633-660

Other

**Conflicts of interest** - Potential sources of influence or perceived influence on study conduct and conclusions; how these were managed	16/696
**Funding** - Sources of funding and other support; role of funders in data collection, interpretation, and reporting	15/675-677

*The authors created the SRQR by searching the literature to identify guidelines, reporting standards, and critical appraisal criteria for qualitative research; reviewing the reference lists of retrieved sources; and contacting experts to gain feedback. The SRQR aims to improve the transparency of all aspects of qualitative research by providing clear standards for reporting qualitative research.

**The rationale should briefly discuss the justification for choosing that theory, approach, method, or technique rather than other options available, the assumptions and limitations implicit in those choices, and how those choices influence study conclusions and transferability. As appropriate, the rationale for several items might be discussed together.

Reference:

O'Brien BC, Harris IB, Beckman TJ, Reed DA, Cook DA. **Standards for reporting qualitative research: a synthesis of recommendations.** *Academic Medicine*, Vol. 89, No. 9 / Sept 2014 DOI: 10.1097/ACM.0000000000000388
